# Supplementary material for: Gene–environment correlations across geographic regions affect genome-wide association studies
Source: Nat Genet. 2022 Aug 22;54(9):1345–54. doi: 10.1038/s41588-022-01158-0 (PMC9470533; doi:10.1038/s41588-022-01158-0)
Supplement: Supplementary file 1 — Supplementary Tables 1–3. [file 41588_2022_1158_MOESM1_ESM.pdf]

nature portfolio

https://doi.org/10.1038/s41588-022-01158-0

Supplementary information

Gene–environment correlations across geographic regions affect genome-wide association studies

In the format provided by the
authors and unedited

Supplementary Tables

Supplementary Table 1: The 56 complex traits and their (effective) sample sizes of their sibling and GWAS analyses.

Category	*Trait*	UK Biobank Field Code	*N-siblings*	*N-GWAS*
**Anthropomorphic:**	Birth weight	20022	25,028	146,855
	Body mass index (BMI)	21001	43,448	253,878
	Body fat percentage	23099	42,889	250,371
	Standing height	50	43,489	254,094
	Hip circumference	49	43,500	254,174
	Waist circumference	48	43,506	254,204
	Whole body fat mass	23100	42,837	250,142
**Cardiovascular**	Diastolic blood pressure, automated reading	4079	41,227	242,738
	Forced expiratory volume in 1-second (FEV1)	3063	40,098	233,983
	Forced vital capacity (FVC)	3062	40,098	233,983
	Mean corpuscular haemoglobin	30050	42,393	247,485
	Mean corpuscular volume	30040	42,393	247,487
	Platelet count	30080	42,393	247,485
	Systolic blood pressure, automated reading	4080	41,227	242,735
**Cognition & SES:**	Educational Attainment	*ISCED*	43,232	252,521
	Financial situation satisfaction	4581	16,554	96,234
	Fluid intelligence score	20016	16,196	94,130
	Household income	738	37,685	218,842
	Work/job satisfaction	4537	11,093	63,780
**Depression:**	Contemplated self-harm	20485	14,308	81,885
	Happiness	4526	16,564	96,266
	Neuroticism score	20127	35,306	207,260
	Sought or received professional help for mental distress	20499	*13,576	*77,630
	Thought that life not worth living	20479	14,270	81,643
**Other Behavior:**	Exceeding motorway speed limit	1100	42,371	248,249
	Moderate physical activity	884	41,439	242,940
	Risk taking	2040	*30,784	*186,025
	Vigorous physical activity	904	43,263	252,961
	Time spent using computer	1080	43,240	252,807
	Time spent watching TV	1070	41,421	243,216
**Physical Health:**	Diabetes diagnosed by doctor	2443	*7,693	*44,957
	Hand grip strength	47	43,413	253,625
	Health satisfaction	4548	16,578	96,337
	Overall health rating	2178	43,394	253,705
**Reproduction:**	Age at first live birth	2754	17,340	95,235
	Age first sexual intercourse	2139	38,203	224,813
	Age at menarche	2714	24,538	133,444
	Age at menopause	3581	15,749	82,301
	Lifetime number of sexual partners	2149	35,878	209,862
	Number of children fathered	2405	18,171	116,528
	Number of live births	2734	25,185	137,177
**Sleep:**	Insomnia	1200	43,516	254,387
	Morning/evening person	1180	38,789	227,250
	Sleep duration	1160	43,320	253,284
	Trouble falling asleep/sleeping too much	20517	14,342	82,030
**Social:**	Been in a confiding relationship as an adult	20522	13,990	80,093
	Family relationship satisfaction	4559	16,580	95,851
	Friendships satisfaction	4570	16,453	95,593
	Loneliness, isolation	2020	*25,240	*142,877
**Substance Use:**	Age at Smoking Initiation	*GSCAN*	14,122	80,561
	Alcohol Frequency	*GSCAN*	43,511	254,383
	Cannabis (ever vs never)	*GSCAN*	14,354	82,092
	Cigarettes per Day	*GSCAN*	13,717	77,651
	Drinks per Week	*GSCAN*	11,703	69,704
	Smoking Cessation	*GSCAN*	19,657	114,069
	Smoking Initiation	*GSCAN*	29,068	172,031

*These are case/control phenotypes for which the effective sample size is reported, which is calculated with: $4/((1/N_{cases})+(1/N_{controls}))$

Supplementary Table 2: Heritability & LDSC intercept of GWASs on height controlled for sex, age, and covariates in the 1st column.

**Covariates**	**Heritability (SE)**	**LDSC intercept (SE)**
100 PCs	0.41 (0.02)	1.31 (0.03)
100 PCs + Lat. & Lon.	0.13 (0.01)	1.69 (0.02)
Lat. & Lon.	0.42 (0.02)	1.68 (0.04)
None	0.44 (0.02)	2.07 (0.04)

Supplementary Table 3: Correlations between latitude, longitude, and the first 5 PCs

	**PC1**	**PC2**	**PC3**	**PC4**	**PC5**
Latitude (birth place)	-.28 ($p<10^{-99}$)	.21 ($p<10^{-99}$)	.02 ($p=10^{-14}$)	.11 ($p<10^{-99}$)	-.13 ($p<10^{-99}$)
Longitude (birth place)	.27 ($p<10^{-99}$)	-.21 ($p<10^{-99}$)	-.08 ($p<10^{-99}$)	.38 ($p<10^{-99}$)	.05 ($p<10^{-99}$)
Latitude (current address)	-.20 ($p<10^{-99}$)	.16 ($p<10^{-99}$)	.005 ($p=.02$)	.11 ($p<10^{-99}$)	-.07 ($p<10^{-99}$)
Longitude (current address)	.21 ($p<10^{-99}$)	-.16 ($p<10^{-99}$)	-.06 ($p<10^{-99}$)	.33 ($p<10^{-99}$)	.04 ($p<10^{-99}$)

Supplementary Table 3 contains Pearson correlations, with their corresponding one-sided p-values, based on 254,557 participants
