# Supplementary material for: Gene–environment correlations across geographic regions affect genome-wide association studies
Source: Nat Genet. 2022 Aug 22;54(9):1345–54. doi: 10.1038/s41588-022-01158-0 (PMC9470533; doi:10.1038/s41588-022-01158-0)
Supplement: Supplementary file 2 — Reporting Summary [file 41588_2022_1158_MOESM2_ESM.pdf]

nature research

Corresponding author(s): Abdel Abdellaoui
Last updated by author(s): Jul 13, 2022

Reporting Summary

Nature Research wishes to improve the reproducibility of the work that we publish. This form provides structure for consistency and transparency in reporting. For further information on Nature Research policies, see our Editorial Policies and the Editorial Policy Checklist.

Statistics

For all statistical analyses, confirm that the following items are present in the figure legend, table legend, main text, or Methods section.

n/a	Confirmed	
☐	☒	The exact sample size (*n*) for each experimental group/condition, given as a discrete number and unit of measurement
☐	☒	A statement on whether measurements were taken from distinct samples or whether the same sample was measured repeatedly
☐	☒	The statistical test(s) used AND whether they are one- or two-sided<br>*Only common tests should be described solely by name; describe more complex techniques in the Methods section.*
☐	☒	A description of all covariates tested
☐	☒	A description of any assumptions or corrections, such as tests of normality and adjustment for multiple comparisons
☐	☒	A full description of the statistical parameters including central tendency (e.g. means) or other basic estimates (e.g. regression coefficient) AND variation (e.g. standard deviation) or associated estimates of uncertainty (e.g. confidence intervals)
☐	☒	For null hypothesis testing, the test statistic (e.g. *F*, *t*, *r*) with confidence intervals, effect sizes, degrees of freedom and *P* value noted<br>*Give P values as exact values whenever suitable.*
☒	☐	For Bayesian analysis, information on the choice of priors and Markov chain Monte Carlo settings
☒	☐	For hierarchical and complex designs, identification of the appropriate level for tests and full reporting of outcomes
☒	☐	Estimates of effect sizes (e.g. Cohen's *d*, Pearson's *r*), indicating how they were calculated

Our web collection on statistics for biologists contains articles on many of the points above.

Software and code

Policy information about availability of computer code

Data collection	No software was used in the data collection.
Data analysis	The open source software used for this study was: GCTA 1.93, PLINK 2.0, flashPCA v2, LDSC Regression 1.0, Genomic SEM 0.0.3, and R 1.4.1717. Our code used for the analyses is available through Zenodo (https://doi.org/10.5281/zenodo.6822023).

For manuscripts utilizing custom algorithms or software that are central to the research but not yet described in published literature, software must be made available to editors and reviewers. We strongly encourage code deposition in a community repository (e.g. GitHub). See the Nature Research guidelines for submitting code & software for further information.

Data

Policy information about availability of data

All manuscripts must include a data availability statement. This statement should provide the following information, where applicable:

- Accession codes, unique identifiers, or web links for publicly available datasets
- A list of figures that have associated raw data
- A description of any restrictions on data availability

This research was conducted using data from the UK Biobank resource (application number 40310). Individual-level UK Biobank data, both phenotypic and genetic, is available to bona fide researchers on request once a research project has been submitted and approved by the UK Biobank committee. Information about registration for access to the data is available at http://www.ukbiobank.ac.uk/register-apply/. The GWAS summary statistics of all 56 complex traits before and after controlling for geographic region are available through Zenodo (https://doi.org/10.5281/zenodo.6822023).

Field-specific reporting

Please select the one below that is the best fit for your research. If you are not sure, read the appropriate sections before making your selection.

☒ Life sciences ☐ Behavioural & social sciences ☐ Ecological, evolutionary & environmental sciences

For a reference copy of the document with all sections, see nature.com/documents/nr-reporting-summary-flat.pdf

Life sciences study design

All studies must disclose on these points even when the disclosure is negative.

Sample size	The sample size was determined by UK Biobank; see for more details Bycroft, C. et al (2018). The UK Biobank resource with deep phenotyping and genomic data. Nature 562, 203.
Data exclusions	In order to avoid bias due to populations stratification, we excluded participants with non-European ancestry as determined by PCA on genome-wide SNPs (details are described in the Online Methods).
Replication	The current study was successful because of the exceptionally large sample size of UK Biobank, allowing for sufficient participants per geographic region in the UK. There are no genotype datasets of similar size and geographic spread as this one, making replication not (yet) feasible.
Randomization	The participants were chosen across 22 assessment centers throughout Great Britain in order to cover a variety of different settings providing socioeconomic and ethnic heterogeneity and urban–rural mix. (see for more details Bycroft, C. et al (2018). The UK Biobank resource with deep phenotyping and genomic data. Nature 562, 203). We investigated the impact of this non-random ascertainmet by including assessment centre as a covariate, and concluded that its impact was not substantial (see our manuscript for more details).
Blinding	Blinding was not relevant as this was not an experimental design, and therefore did not have experimental groups; we included everyone in our analyses from this population-based sample with genotype and phenotype data available.

Reporting for specific materials, systems and methods

We require information from authors about some types of materials, experimental systems and methods used in many studies. Here, indicate whether each material, system or method listed is relevant to your study. If you are not sure if a list item applies to your research, read the appropriate section before selecting a response.

Materials & experimental systems

n/a	Involved in the study	
☒	☐	Antibodies
☒	☐	Eukaryotic cell lines
☒	☐	Palaeontology and archaeology
☒	☐	Animals and other organisms
☐	☒	Human research participants
☒	☐	Clinical data
☒	☐	Dual use research of concern

Methods

n/a	Involved in the study	
☒	☐	ChIP-seq
☒	☐	Flow cytometry
☒	☐	MRI-based neuroimaging

Human research participants

Policy information about studies involving human research participants

Population characteristics	A total of 502,536 participants (273,402 females and 229,134 males) aged between 37 and 73 years old were recruited in the UK between 2006 and 2010. The participants were recruited across 22 assessment centers throughout Great Britain in order to cover a variety of different settings providing socioeconomic and ethnic heterogeneity and urban–rural mix. This is a population-based sample, wherein participants were not chosen based on current or past diagnoses. See for more details Bycroft, C. et al (2018). The UK Biobank resource with deep phenotyping and genomic data. Nature 562, 203.
Recruitment	The UK Biobank ascertainment strategy was designed to capture sufficient variation in socioeconomic, urban–rural, and ethnic background. The participation rate however was 5.45% and was biased towards older, more healthy, and female residents. The UK Biobank sample does reflect nationally representative data sources to a significant degree.
Ethics oversight	The participants of this study come from UK Biobank (UKB), which has received ethical approval from the National Health Service North West Centre for Research Ethics Committee (reference: 11/NW/0382).

Note that full information on the approval of the study protocol must also be provided in the manuscript.
